# An Unexpected Finding of Isolated Hydatidosis of the Spleen With Concomitant Visceromegaly in an Elderly Female

**DOI:** 10.7759/cureus.46346

**Published:** 2023-10-02

**Authors:** Aditya Sharma, Ganesh Chandra Yadav, Amit Kumar Yadav, Ram Niwas Meena

**Affiliations:** 1 Department of General Surgery, Institute of Medical Sciences, Banaras Hindu University, Varanasi, IND; 2 Department of General Surgery, Heritage Institute of Medical Sciences, Varanasi, IND; 3 Department of General Surgery, Maa Vindhyavasini Autonomous State Medical College, Mirzapur, IND

**Keywords:** splenic hydatidosis, splenomegaly, zoonotic disease, splenectomy, rare presentation

## Abstract

Hydatidosis of the spleen is a rare zoonotic clinical entity. The occurrence of isolated splenic hydatid cysts in the absence of these cysts in any other portion of the body is referred to as primary splenic hydatidosis. It is a rare disorder that accounts for only 2% of the burden of hydatid disease worldwide. After the liver and the lungs, the spleen is the organ that is most frequently affected by this condition.

## Introduction

The first person to describe splenic hydatidosis as an autopsy finding was Berlot in 1790 [1]. Countries like the Middle East, India, Australia, South America, and New Zealand all have high rates of the disease [2]. It is a parasitic infestation caused by the tapeworm *Echinococcus granulosus* that results in the hydatid disease. *Echinococcus granulosus* infestations are common, particularly in communities where sheep, dogs, and other cattle are kept in close proximity to people. The parasite lives inside dogs and other canine family members [3]. The mainstay of treatment is surgery, i.e., a partial or total splenectomy.

## Case presentation

A 60-year-old female with chief complaints of pain and a dragging sensation in the abdomen for the past two years presented to the surgery emergency department. The pain was confined to the upper quadrants of the abdomen and was constant, dull-aching in character, and not radiating. There was no history of fever, weight loss, or loss of appetite. A history of trauma to the abdomen was absent.

When examined, she was afebrile and pallid rather than icteric or cyanotic. Her blood pressure (BP) was 116/70 mmHg, and her pulse rate (PR) was 82/min. Her cardiac and pulmonary systems were within normal limits after a systematic assessment. Per abdomen examination, the abdomen was tense with mild discomfort in the epigastric and left hypochondrium regions.

On abdominal ultrasonography (USG), a well-defined multicystic lesion measuring 27.5x16.0 cm with notable internal splenic septations was identified along with the visceromegaly. Contrast-enhanced computed tomography (CECT) revealed gross enlargement of the spleen with multiple hypo-dense non-enhancing lesions of varying sizes, mostly in the splenic parenchyma, and most of them with peripheral calcified rim features suggestive of hydatid disease of the spleen. There was mild hepatomegaly but no hepatic involvement.

**Figure 1:**
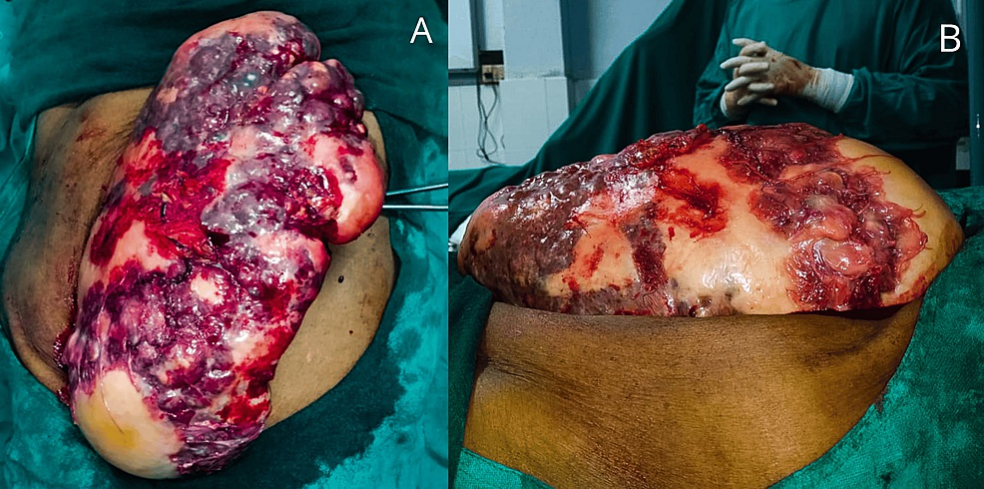
An intraoperative picture showing the giant spleen covering almost the whole abdomen. (A) Anteroposterior view and (B) oblique view

**Figure 2:**
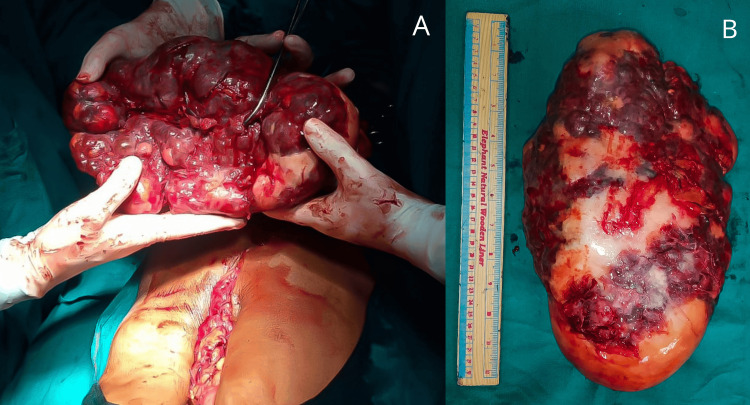
A cut resected specimen of the spleen. (A) Intraoperatively with 18 cm laparotomy wound. (B) Postoperatively measuring around 30x14 cm.

She was started on albendazole oral administration at a dose of 15 mg/kg/day in two daily doses for four weeks. The patient was planned for open total splenectomy on an elective basis, and preoperative vaccination with pneumococcal polysaccharide conjugate vaccine (adsorbed) IP, 13-valent inactivated influenza vaccine (split virion) IP, and meningococcal (groups A, C, Y, and W-135) polysaccharide diphtheria toxoid conjugate vaccine was done. Intraoperatively, a giant spleen measuring 30x14 cm was noted, as shown in Figure 1, and was resected after removing all the ligament attachments, as shown in Figure 2.

The histopathology of the resected specimen came out to be a hydatid disease of the spleen. The postoperative period was uneventful, and the patient was allowed oral intake on postoperative day two and discharged on day four, and she did well in her follow-up visits.

## Discussion

The isolated hydatid disease is a very rare presentation, with less than 2% of the cases reported among all such cases. Humans are susceptible to infection through direct contact with dogs or by consuming food or liquid that has been contaminated with dog bodily waste [4]. The liver and lungs are the organs most frequently infected by the parasite, followed by the kidneys, brain, and bones. The spleen, pancreas, and muscles are the organs that are very rarely affected; the spleen is involved in 5% of instances, and isolated involvement is seen in the rarest of rare case scenarios [5].

In cases of cystic lesions in the spleen in endemic locations, the primary splenic hydatid cyst should be kept as a differential diagnosis unless otherwise proved [6]. However, partial splenectomy and other spleen-preserving procedures are advised in cases with superficial cysts, cysts localised in one pole, cases of significant adhesions, and in infants [7]. Total splenectomy is the best course of treatment for enormous, central splenic hydatid cysts involving the hilar area [8]. The risk of severe post-splenectomy infections and recurrences should be minimised by recommending perioperative vaccinations and albendazole medication.

## Conclusions

Even though splenic hydatid disease is uncommon, a diagnosis can typically be made based on the patient's history, symptoms, and radiological examination using an abdomen USG or CT scan. A serologic test, abdominal exploration either open or laparoscopic, percutaneous aspiration, or biopsy may all be employed to establish the diagnosis.

## References

[1] Rasheed K, Zargar SA, Telwani AA (2013). Hydatid cyst of spleen: a diagnostic challenge. N Am J Med Sci.

[2] King CH (1995). Cestodes (tapeworms). Mandell, Douglas and Bennett’s Principles and Practice of Infectious Disease.

[3] Moro P, Schantz PM (2009). Echinococcosis: a review. Int J Infect Dis.

[4] Moguillanski SJ, Gimenez CR, Villavicencio RL (1999). Radiology of abdominal hydatidosis. Radiology and Diagnostic and Therapeutic Imaging: Abdomen.

[5] Beggs I (1985). The radiology of hydatid disease. AJR Am J Roentgenol.

[6] de Diego J, Lecumberri FJ, Franquet T, Ostiz S (1982). Computed tomography in hepatic echinococcosis. AJR Am J Roentgenol.

[7] Marani SA, Canossi GC, Nicoli FA, Alberti GP, Monni SG, Casolo PM (1990). Hydatid disease: MR imaging study. Radiology.

[8] Franquet T, Montes M, Lecumberri FJ, Esparza J, Bescos JM (1990). Hydatid disease of the spleen: imaging findings in nine patients. AJR Am J Roentgenol.

